# Effect of Addition of Thyroxine in the Treatment of Graves’ Disease: A Systematic Review

**DOI:** 10.3389/fendo.2020.560157

**Published:** 2021-01-25

**Authors:** Jun Li, Litao Bai, Fan Wei, Maoying Wei, Yao Xiao, Weitian Yan, Junping Wei

**Affiliations:** ^1^ Department of Endocrinology, Guang’anmen Hospital, China Academy of Chinese Medical Sciences, Beijing, China; ^2^ The Second Affiliated Hospital of Chongqing Medical University, Chongqing, China

**Keywords:** Graves’ disease, hyperthyroidism, treatment, thyroxine, review

## Abstract

Graves’ disease is the most common cause of hyperthyroidism. Antithyroid drugs, radioiodine ablation, and surgery are the main treatments. Research has demonstrated that adding thyroxine to antithyroid therapy can improve the remission rate, and many similar studies have been conducted subsequently. The purpose of this systematic review was to investigate whether adding thyroxine to various treatments for Graves’ disease has a clinical benefit in remission/relapse rate, stable thyroid function, occurrence of Graves’ ophthalmopathy, etc. A total of 27 studies were included, and the risk of research bias was moderate to high. We discuss the role of thyroxine both in pharmacological and non-pharmacological therapeutic regimens. Overall, the available evidence does not support the indiscriminate addition of thyroxine to various treatments for Graves’ disease, especially in combination with oral antithyroid drugs. Further clinical studies are required to explore the indications of thyroxine addition in the treatment of Graves’ disease.

## Introduction

Hyperthyroidism is characterized by increased thyroid hormone synthesis and secretion. The most common cause of hyperthyroidism is Graves’ disease, followed by the toxic multinodular goiter. The worldwide prevalence of hyperthyroidism varies by region. A meta-analysis of studies that evaluated the prevalence and/or incidence of thyroid dysfunction in Europe showed a prevalence of about 0.8%, while the National Health and Nutrition Examination Survey in the United States showed a prevalence of 1.3% (1, 2). Global variations in the epidemiology of hyperthyroidism may be due to differences in diagnostic thresholds, assay sensitivity, population selection, and iodine nutritional status (3). In areas with adequate iodine intake, most thyroid dysfunctions are due to thyroid autoimmunity. Numerous studies have revealed the effects of changes in iodine status and the epidemiological effects of iodine supplementation on thyroid dysfunction (4, 5). Studies in Denmark have shown that Graves’ disease accounts for 70–80% of patients with hyperthyroidism in areas with adequate iodine intake, while in areas with iodine deficiency it accounts for about 50% of cases (6). The etiology of Graves’ disease is considered multifaceted because of the reduced immune tolerance and the development of autoantibodies that stimulate thyroid follicular cells by binding to the thyroid-stimulating hormone (TSH) receptor. There is evidence of the genetic susceptibility of Graves’ disease (7). Among non-genetic factors, iodine nutritional status, psychological stress, gender, smoking, low selenium, immunomodulators, etc., may contribute to the progress of the disease (8–13). Hyperthyroidism treatments include antithyroid drugs (ATD), radioactive iodine ablation, and surgery. Outside the United States, ATD are preferred as primary treatment, while surgery and radioactive iodine are utilized in patients with persistent or recurrent hyperthyroidism (14, 15 **).** There are two ways to use ATD: titration, and block & replace. With titration, the dose of ATD is titrated over time to the lowest dose needed for maintaining an euthyroid state. In the block-and-replace regimen, a higher dose of ATD is used with concurrent replacement with levothyroxine. A meta-analysis including twelve studies in 2010 investigated the effects of B&R and titration block protocols. Although there were adverse reactions, the recurrence rate was the same in the two groups, 51% in the B&R group and 54% in the titration block group (odds ratio, 0.86; 95% confidence interval, 0.68 to 1.08). Withdrawal due to side effects (16% and 9%, respectively) was significantly greater in the B&R group (16 **).** Although the block-and-replace regimen is controversial in many studies, it is still used in many regions.

The role of thyroxine in the treatment of Graves’ disease needs to be evaluated on multiple aspects. The purpose of this study was to examine the addition of thyroxine for the treatment of Graves’ disease and explore its benefits and deficiencies. This study was designed as a systematic review.

## Methodology

### Criteria for Considering Studies for This Review

#### Types of Study

Randomized controlled trials (RCT), non-RCT, crossover studies, retrospective studies, and comparative studies were included in this analysis. Protocols and case reports were ruled out. Abstracts and non-English language publications were equally excluded.

#### Type of Participants

We included patients with a clinical diagnosis of Graves’ disease. There were no restrictions on the age or gender of patients.

#### Type of Interventions and Comparisons

We included studies that combined thyroxine in the treatment of Graves’ disease, where the primary endpoint was the remission/relapse rate of Graves’ disease with and without thyroxine. Other effects of this combination therapy in the treatment of Graves’ disease were also considered for demonstration in this study, including stable thyroid function, occurrence of Graves’ ophthalmopathy, quality of life, operative process, postoperative complications and so on. If patients with Graves’ disease developed hypothyroidism and used thyroxine, the respective studies were excluded.,

#### Search Strategy

We searched the following publication databases: PubMed, Embase, Cochrane Library, and Web of Science. Studies published in English before December 31, 2019, were included. Selected keywords included Graves’ disease, Basedow’s disease, thyroxine, levothyroxine, L-thyroxine. Keywords were customized for each scientific database. The references of all retrieved articles were examined to ensure that all relevant articles were included for data synthesis. The retrieval strategy and process are illustrated in the **Supplementary Material**.

### Data Collection and Analysis

#### Selection of Studies

Two authors (J.L. and L.B.) independently reviewed all potential studies for inclusion against the eligibility criteria. They examined the title and abstract and, where necessary, the full text of the studies to assess if they were eligible for inclusion. In cases where they were not able to reach an agreement, a third author (W.Y.) made the final decision concerning eligibility.

#### Data Extraction

Two authors, M.W. and Y.X., independently assessed and selected studies eligible for this systematic review based on their title and abstract. Disagreement was resolved by consensus. Two authors, J.L. and L.B., read the full texts of the remaining articles and made a final selection. Data were extracted and presented in a table, including author, year, country, study design, sample, sex ratio (male-to-female), age (years), medication, intervention process, follow-up, assessments, results, side effects.

#### Assessment of Risk of Bias

The included studies were evaluated for the risk of bias using the Cochrane Collaboration’s tool for randomized trials. The following aspects of the study were evaluated: random sequence generation, allocation concealment, blinding of participants and personnel, blinding of outcome assessment, incomplete outcome data addressed (intention-to-treat analysis), selective reporting, other bias. The non-RCT studies were assessed using the MINORS score, which serves as a methodological index for non-randomized studies. It has 12 domains for which non-comparative studies use the first 8 domains. Each domain is scored out of 2 with ideal scores being at least 16 for non-comparative studies and 24 for comparative studies.

## Results

**Figure 1:**
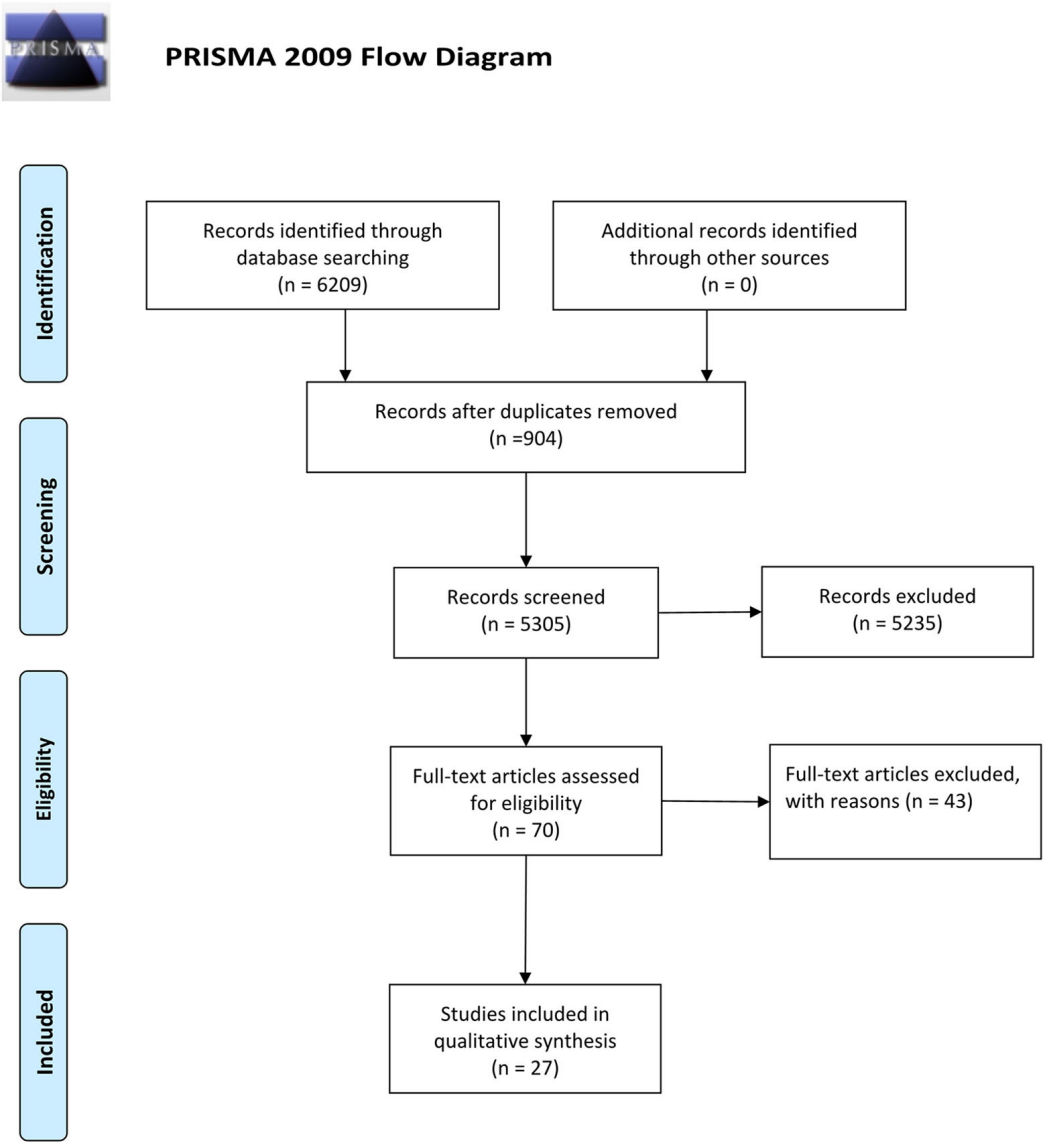
Flow diagram.

**Figure 2:**
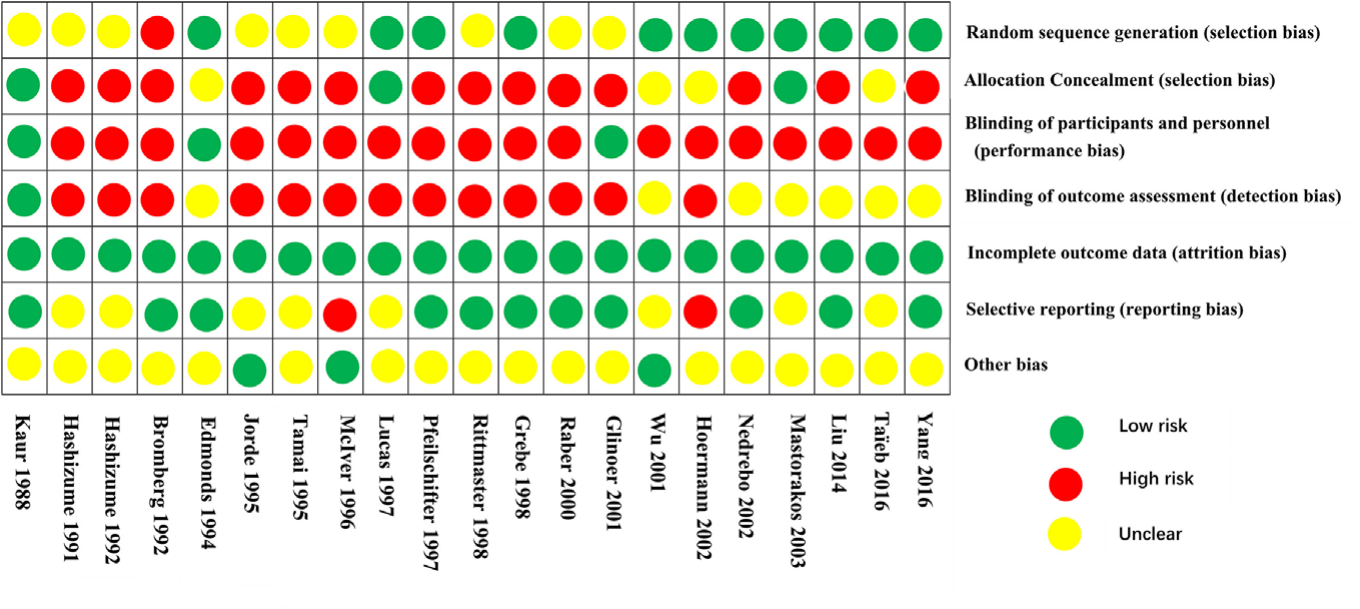
Risk of bias assessment of randomized controlled trials.

**Figure 3:**
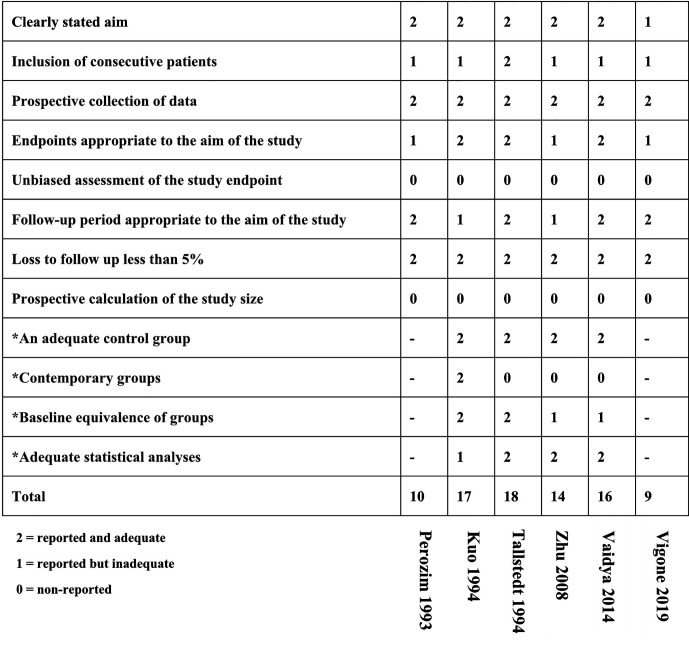
Quality assessment according to the MINORS assessment tool.

The search resulted in 6,209 potential articles from PubMed (n = 1,777), Cochrane Library (n = 95), Embase (n = 3,672), and Web of Science (n = 665). In total, 6,139 articles were excluded based on the double-blind screening of the title and abstract, including 904 duplicates. The remaining 70 full text articles were assessed, and 27 articles were found eligible for this review (21 RCT and 6 non-RCT). **Figure 1** shows the flowchart of the search and selection process. Overall, the risk of bias of randomized studies was moderate to high, as shown in **Figure 2**. The MINORS score of non-RCT studies were assessed in **Figure 3**. Research of Graves’ disease related to pharmacotherapy is shown in **Tables 1**–**3**, while non-pharmacological research is shown in **Table 4**.

**Table 1:** Thyroxine in the “Block-and-Replace” regimen.

Study	Country	Design	Sample	Sex(M:F)	Age(yrs)	Medication	Intervention process	Follow-up		Assessments	Results	Side effect
Bromberg et al. (17)	Brazil	RCT	45	A(4:28)/B(2:12)	A(range 17–55 years)/B(range 21–49 years)	A (40–100mg MMI or 400–900mg PTU+75ug T3)/B(5–25mg MMI or 50–300mg PTU)	duration of therapy was similar for both groups: range 12–26 months for group A, range 10–28 months for group B	before. during treatment and 5–6 weeks after withdrawal of ATD	T3, T4, TSH, FT4, TgAb, eye signs		no obvious difference in the improvement of Graves’ ophthalmopathy between the two treatment options	–
Perozim et al. (18)	Brazil	Non-RCT	28	4:24	14–53	40mg MMI + 75ug T4	60mg MMI (4–6 weeks) → 40mg MMI+ 75ug T4 (18–24 months)	18–24 months MMI+L-T4 → additional 2 years after treatment	ultrasonographic, TBG, TRAb, T3, T4		decreases TRAb and the frequency of recurrence of hyperthyroidism	–
Edmonds and Tellez (19)	UK	RCT	70	HD(6:28)/TD(11:25)	HD(48 ± 11.9)/TD(41 ± 12.9)	HD (60mg Carb. +100–150mg T4)/TD (60mg Carb. → maintenance dose Carb.)	HD (4 weeks Carb. →1 year Carb. +T4), TD (4 weeks Carb. →1 year maintenance dose Carb.)	2 years after stopping treatment (monthly→ 3-month intervals)	FT3, TSH, TRAb, TBG, pertechnetate uptakes, ophthalmopathy		measured variables and progress of patients was similar in the HD or TD regimen	HD (8), TD (6)
Jorde et al. (20)	Norway	RCT	56	HD(5:24)/LD(6:21)	HD(40.6 ± 2.1)/LD(43.8 ± 2.8)	HD (60mg MMI+0.1–0.15mg T4)/LD (30mg MMI)	HD (2–4 weeks 60mg MMI→MMI+0.1–0.15mg T4), LD (30mg MMI→ dose titrated every 2–4 weeks), all treatment stop after 6 months	every 3 months (first 1 year) →every 6 months (the 2 year)	thyroid volume, T3, T4, TSH, eye symptoms		significantly reduced the relapse rate the 1 year, the relapse rates in both groups were unacceptably high	HD (9), LD (4)
McIver et al. (21)	UK	RCT	111	A(11:41)/B(11:48)	A(33 ± 9)/B(36 ± 10)	A(maintenance dose Carb.)/B (40mg Carb. + 100ug T4)	40mg Carb. (1 month)→group A (Carb. alone 17 months), group B (17 months Carb. +T4→T4 alone 18 months)	median follow-up period was 12 months	T3, T4, TSH, TRAb		neither delays nor prevents the recurrence of hyperthyroidism	5(urticaria, arthralgia, nausea)
Lucas et al. (22)	Spain	RCT	60	A(7:23)/B(4:26)	A(37.5 ± 13.9)/B(34.5 ± 8.3)	A(maintenance dose Carb.)/B(30–45mg Carb.+75–150ug L-T4)	45–60 mg Carb.→divided into 2 groups, all treatment during 12–24 months	3-month interval (first 2 years)→annually thereafter, or when relapse	T3, T4, FT4, TSH, TBG, goiter size, ophthalmopathy		offers no advantages in the treatment of GD	–
Grebe et al. (23)	New Zealand	RCT	37	H(5:12)/L(3:17)	H(33 ± 8.7)/L(33.7 ± 11.9)	HD (100mg Carb. +T4)/LD (25mg→titrating dose Carb.)	all treatment discontinue after 6 months	every 6 weeks (first 3 months) → 3-monthly (first year) →6-monthly (the 2 year)	FT4, FT3, TSH, IgA, IgG, IgM, WBC, RBC		delays, but not prevent relapse	HD(7), LD(1)
Nedrebo et al. (24)	Norway	RCT	218	1A(0.148:0.152)/1B(0.161:0.839)/2A(0.074:0.926)/2B(0.166:0.834)	1A(43.9 ± 10.2)/1B(40.2 ± 11.5)/2A(42.4 ± 12.2)/2B(43.4 ± 13.3)	1A(Carb.+L-T4→L-T4)/1B (Carb.+L-T4→ no drugs)/2A (Carb.→L-T4)/2B(Carb.→ no drugs)	Carb. (12 months): group 1 (B&R therapy) and group 2 (titration regimen) →divided into 2 subgroups (1 year)	before treatment→ after 3 and 6 weeks→ every 3 months	TRAb, TSH, FT4, T3, size of goiter		relapse rates were independent of Carb. regimen whether followed by L-T4 therapy or not	rash (21)
Vaidya et al. (25)	UK	Non-RCT	450	B&R(20.2:79.8)/Titration(13.4:86.6)/Both(17.9:82.1)	B&R(49 ± 15)/Titration(49 ± 18)/Both(48 ± 15)	B&R/Titration/Both titration and B&R	retrospectively examined case-records of patients with GD	when relapses (only included the first episode), treated more than 2 years (included the first 2 years)	FT4, FT3, TSH, TRAb, TPOAb		little evidence that patients under B&R have more stable thyroid function	skin rash (1.3%:6%), neutropenia (1.3%:0.7%), liver dysfunction (0%:0.7%)

**Table 2:** Thyroxine addition in the titration regimen.

Study	Country	Design	Sample	Sex(M:F)	Age(yrs)	Medication	Intervention process	Follow-up	Assessments	Results	Side effect
Hashizume et al. (26)	Japan	RCT	109	A1(7:22)/A2(6:21)/B1(7:24)/B2(4:18)	A1(36 ± 6)/A2(32 ± 7)/B1(33 ± 7)/B2(34 ± 6)	A1(100μg T4 +10mg MMI)/A2(placebo+10mg MMI)/B1 (same as A1)/B2(same as A2)	30mg MMI (6 months) →divided into Group A (antibody level 15% or more) or B (antibody level less than 15%) → divided into 2 subgroups	stop MMI within 1 year after MMI+T4 therapy → T4 or placebo alone for 3 years	T3, T4, TSH, TBG, TRAb	decreases production of TRAb and relapse rate	not found
Kuo et al. (27)	China	Non-RCT	60	A(6:19)/B(5:10)/C(4:16)	A(34 ± 5)/B(37 ± 6)/C(36 ± 6)	A (10mg MMI +0.1mg T4)/B (10mg MMI)/C (10mg MMI)	30mg MMI (3 months) →15mg MMI (3 months) →divided into 3 groups (6 months)	6 months and 12 months of combination therapy	FT4, TSH, TRAb, TBG, T3, T4, thyroid volume	patients treated with MMI+T4 have greater decline in TRAb and TBG	–
Tamai et al. (28)	Japan	RCT	105	A(8:27)/B(9:26)/C(8:27)	A(35.3 ± 2.4)/B(36.8 ± 2.3)/C(36.2 ± 2.3)	A((maintenance MMI+75–100ug T4, TSH normal))/B(same as A, TSH suppressed)/C((maintenance dose MMI)	MMI (1 year)→divided into 2 groups→group 1:MMI+ T4/group 2: MMI (6 months) →group 1 divided into group A and B, group 2 as groupC(6 months)	12 months after treatment	TSH, T4, FT4, FT3, TBG, TRAb	no effect on TRAb and rates of recurrence	–
Pfeilschifter and Ziegler (29)	Germany	RCT	50	A(2:8)/B(3:9)/C(7:21)	A(36.0 ± 15.1)/B(39.6 ± 13.3)/C(41.1 ± 10.2)	A (10mg Carb.+ low dose T4)/B(10mg Carb.)/C(10mg Carb.+ 50–150ug T4→T4)	3 groups for 12 months treatment→ ATD discontinued after 1 year→suppressive doses T4 continued another year	3-month intervals	TSH, T3, T4, TBG	unable to detect a preventive effect on the recurrence of hyperthyroidism, benefit for thyroid enlargement	not found
Rittmaster et al. (30)	Canada	RCT	149	1(15:85)/2(14:86)/3(10:90)	1(38 ± 14)/2(39 ± 14)/3(38 ± 13)	1(30mg MMI →maintenance dose MMI)/2(30mg MMI→30mg MMI+ T4→T4)/3(30mg MMII→30mg MMI+T4→T4)	divided into 3 groups for 18 months: group 1(maintenance dose MMI)/group 2(MMI+T4) to maintain TSH 2.0–5.4mIU/L/group 3(MMI+T4) to maintain TSH ≤0.6mIU/L→T4 continue in group 2 and group 3(TSH in low-normal range or below normal)	3–4 week intervals(first 6 months) →every 2 months(6–18 months) →after methimazole stop 2 or 3 weeks to identify whether remission →if remission, every 1–2 months(next 2 yrs) → every 6–12 months	FT4, FT3, TSH, TRAb, thyroid size	addition of T4 to MMI does not improve long-term remission rates	rash and/or itching(41), joint or muscle discomfort(31), metallic taste(45), gastrointestinal complaints(9), jaw pain(2), agranulocytosis(1), hepatitis(2)
Raber et al. (31)	Austria	RCT	114	1(11:89)/2(16:84)/3(18:82)	1(40 ± 13)/2(41 ± 11)/3(43 ± 13)	group 1(MMI→ stop MMI)/group 2(MMI→10mg MMI+50ug- variable dose T3)/group 3(MMI→50ug- variable dose T3)	60mg MMI for 1 week →40mg MMI for 1 week→20mg for week 3→adjust MMI on thyroid function (8.5 ± 7.4 weeks) → maintenance dose MMI (9.0 ± 2.5 months) →divided into 3 groups for 6 months: group 1(stop MMI)/group 2(MMI+T3) to suppress TSH <0.1mU/L/group 3(T3) to suppress TSH <0.1mU/L	every 3 weeks after diagnosis→ every 4–6 weeks throughout the study after divided into 3 groups (mean follow-up of 16 months)	T3, T4, FT4, TSH, TRAb, thyroid size, 24h urinary iodine excretion	no significant difference was seen in relapse	Palpitations (7), light-headedness (1), diarrhea (1), weight instability (1)
Glinoer et al. (32)	Belgium	RCT	82	12.15:70.85	mean age of 36 years	A (30–80mg ATD →25mg ATD+L-T4→L-T4)/B (30–80mg ATD → 25mg ATD+L-T4→no drugs)	Phase I: 30–80mg ATD (1 month) → 25mg ATD+85ug L-T4(4 month) → 25mg ATD+100ug L-T4(6 month) → 25mg ATD+110ug L-T4(9 month) → 25mg ATD+115ug L-T4(15 month) Phase II: randomly assigned into 2 group, A (100ug L-T4 for 12 months)/B (placebo)	Phase I: 1 (optional), 2, 4, 6, 9, 12, 15 months Phase II: every 3 months	FT4, FT3, TSH, TRAb	L-T4 administration during and after ATD withdrawal did not improve remission rate	–
Wu et al. (33)	China	RCT	60	A(4:16)/B(5:15)/C(5:15)	A(35 ± 7)/B(39 ± 6)/C(35 ± 5)	A(5–10mg MMI +50ug L-T4→50ug L-T4)/B(5–10mg MMI→5mg MMI)/C(5–10mg MMI +50ug L-T4→5mg MMI+50ug L-T4)	30mg- maintenance dose MMI (6 months) → distributed into 3 groups (24 months) → A (6 months L-T4 alone), B (6 months MMI), C(6 months MMI+L-T4)	every 3 months within 1 year of discontinuation	FT3, FT4, TSH, TSAb	cannot reduce the recurrence rate more effectively than with only MMI	–
Hoermann et al. (34)	Germany	RCT	225	A(13:98)/B(16:98)	A(41 ± 1.87)/B(41 ± 1.10)	A(no drugs)/B(maintenance dose L-T4)	ATD (12–15 months) →divide into 2 groups for 1 year (4 weeks after drug withdrawal) →treatment based on test results (1 year)	1, 3, 6, 12, 18, 24 months	FT3, FT4, TSH, T3, T4, TRAb, thyroid volume	not prevent relapse of hyperthyroidism after restoration of euthyroid function by ATD	–
Mastorakos et al. (35)	Greece	RCT	108	T4(6:27)/T3(8:30)/placebo (8:29)	T4(43.8 ± 11.6)/T3(42.6 ± 15.2)/placebo(43.1 ± 13.8)	T4(100μgT4)/T3(25μg T3)/placebo	ATD (24 months), assignment into 3 groups during the period	evaluated trimonthly up to 12 months and at 24 months.	TT3, TT4, FT3, FT4, TSH, TRAb, TPOAb, TgAb, thyroid weight, exopthalmos	T4 administration after ATD treatment is associated with increased recurrence compared to T3 or placebo	–
Liu et al. (36)	China	RCT	145	Group 1(7:39)/Group 2(6:41)/Group 3(9:43)	Group 1 (33.1 ± 10.9)/Group 2 (33.1 ± 12.3)/Group 3 (34.3 ± 13.9)	Group 1(30mg →5mg MMI)/Group 2 (30mg →15mg MMI→ 10mg MMI +L-T4→ 5mg+L-T4)/Group 3(30mg →10mg MMI→ 10mg+L-T4→ 5mg+L-T4→ 2.5mg+L-T4)	30mg MMI (at least 1 month) → treat in 3 groups for 5 months (MMI, 30mg→20mg→15mg→10mg→5mg, add L-T4 when appropriate)	1, 3, 6, 9, 12, 18, 24, 36, 48, 60 and 72 months after drug withdrawal	FT4, FT3, TSH, thyroid size	addition of L-T4 to MMI neither improves nor prevents the remission or recurrence	–
Yang et al. (37)	China	RCT	109	C(16:38)/I(17:38)	C(64.49 ± 3.39)/T(63.87 ± 3.16)	C(15–30 mg MMI → maintenance dose MMI)/T(15–30mg MMI +25ug L-T4→ maintenance dose MMI+25ug L-T4)	both groups treat for 18 months	after completion of treatment	TSH, FT3, FT4, TRAb, ALP, CT, 24h UP, 24h UCa, PTH, blood calcium, serum phosphorus, BMD	beneficial effect on bone metabolism in older patients, no difference in remission rates	–

**Table 3:** Thyroxine addition in special populations.

Study	Country	Design	Sample	Sex(M:F)	Age(yrs)	Medication	Intervention process	Follow-up		Assessments	Results	Side effect
Hashizume et al. (38)	Japan	RCT	78	pregnancy	A (25.8 ± 4.1)/B (26.2 ± 4.2)	A (100ug T4)/B (no drugs)	euthyroid 2 months before pregnancy (10–30mg MMI for 1–3yr)→MMI discontinued 5–6 months after onset of pregnancy →divide into 2 groups:A (T4 continue for 1yr after delivery)/B(no drug)	5–6 months after onset of pregnancy until 1 year after delivery	T3, T4, TBG, TSH, TRAb		decreases TRAb and prevent the postpartum recurrence	–
Vigone et al. (39)	Italy	Non-RCT	28	4:24	average age is 9.2 years (ranging from 2.58 to 16.83 years)	MMI (average 0.3mg/kg/day) + L-T4 (average dose of 1.12μg/kg/day)	ATD (1.5 years) → poor control of thyroid function→ combination therapy for 2.85 years (ranging from 5 months to 7 years)	2–4 weeks (first 6 months) →2–4 months, in case of remission (2–4 months in first 2 years→6–12 months)	ultrasonography, TSH, FT3, FT4, TT3, TT4		better control of thyroid function, delay radioiodine treatment for 4.9 years and surgery for 2.9 years, not increase remission rates	neutropenia (1)

**Table 4:** Non-pharmacological research of Graves’ disease.

Study	Country	Design	Sample	Sex(M:F)	Age(yrs)	Medication	Intervention process	Follow-up	Assessments	Results	Side effect
Kaur et al. (40)	UK	RCT	34	Group 1(3:9)/group 2 (3:7)/group3 (3:9)	Group 1 (38y11 m ±8y1m)/group 2(34 y8 m ± 10y10 m)/group 3(32y 11m ±8y)	Group 1 (I_2_ alone)/group 2 (ATD+T_4_)/group 3((ATD+ T_4_+I_2_)	use ATD for 4–6 weeks until euthyroid →add L-T4 0.15–0.2mg. for 2–3 months →divide into 3 groups for 10 days preoperatively	1 day preoperatively → the day after→ 5 days after operation	size of goiter, T4, FT4, vascularity of gland, operative blood loss, thyroid follicular size	addition of iodine preoperatively is unnecessary in the patient who is euthyroid on ATD and L-T4	–
Tallstedt et al. (41)	Sweden	Non-RCT	492	A(20:80)/B(14:86)	A(60 ± 13)/B(59 ± 12)	A (maintenance dose T4)/B (0.05mg→0.1mg T4)	^131^I therapy →group A received T4 (when hypothyroidism), group B received 0.05mg of T4(2 weeks after ^131^I) and 0.1mg (after further 2 weeks)	18 months after therapy	size of thyroid, T3, T4, TSH, soft-tissue involvement, Optic nerve damage	early administration of T4 after ^131^I reduces the occurrence of Graves’ ophthalmopathy	–
Zhu et al. (42)	China	Non-RCT	168	C(82)/I(A23/B 21/C 25/D 17)	C(-)/I(-)	Control (MMI or PTU)/A (MMI or PTU)/B(same as A)/C(MMI or PTU)/D(same as C)	45–90mg MMI or 45mg–900mg PTU→1.6pg/kg L-T4 or 40–60mg thyroid pills while continuing ATD (A/C is 2 months, B/D is 1 months)	1 month (first 3 months) →every 3 months(1 year)	FT4, FT3, TSH, symptom rating Scale, thyroid crisis, injury to RLN, low calcium, bleeding	sequential thyroid defunctionalization followed by thyroxine supplementation reduce the bleeding volume and postoperative complication rate	–
Taïeb et al. (43)	European	RCT	94	group A(12:34)/group B(11:37)	group A (48.8 ± 13.5)/group B (47.1 ± 12.9)	group A(early prophylactic L-T4 treatment)/group B (L-T4 when needed)	group A(50µg L-T4, 5 days post-radioiodine→ dose adapted at 1, 3 and 6 months), group B(followed up every 4 weeks and treated with LT4 when needed)	1, 3, 6,12 months post-RAI	FT4, FT3, TSH, TRAb, ThyPRO, QoL	Early LT4 administration post- RAI represent potential benefit for QoL. RAI activities and LT4 treatment dosage and timing remains to be determined	not reported

ALP, alkaline phosphatase; ATD, antithyroid drugs; Carb., carbimazole; FT3, serum free triiodothyronine; FT4, free thyroxine; GD, graves’ disease; HD, high dose; HR, heart rate; HRQL, hypothyroid-health related quality of life; IL-6, interleukin-6; IL-8, interleukin-8; IL-10, interleukin-10; IgA, immunoglobulin A; IgG, immunoglobulin G; IgM, immunoglobulin M; LD, low dose; L-T4, levo-thyroxine; MAP, mean artery pressure; MMI, methimazole; PTH, parathyroid hormone; QoL, quality of life; RBC, red blood cells; RLN, recurrent laryngeal nerve; SBP, systolic blood pressure; T3, triiodothyronine; T4, thyroxine; TBG, thyroxine binding globulin; TgAb, thyroglobulin antibody; ThyPRO, thyroid specific patient-reported outcome; TPOAb, thyroid peroxidase antibody; TRAb, thyrotropin receptor antibodies; TSAb, thyroid stimulating antibody; TSH, thyroid stimulating hormone; TSQ, thyroid specific questionnaire; Uca, urine calcium; UP, urine phosphorus; WBC, white blood cell.

### Effects of Thyroxine Addition on the Relapse or Remission Rates of Graves’ Disease

#### Thyroxine in the “Block-and-Replace” Regimen

The “block-and-replace” (B&R) regimen is well known to endocrinologists, but it is not routinely recommended in clinical practice. The B&R regimen completely blocks the thyroid hormone synthesis with high-dose ATD, while supplementing exogenous thyroxine to replace normal thyroid function. As per the 2016 American Thyroid Association guidelines, B&R therapy should be avoided or, if necessary, should be used after addressing compliance, in patients whose condition was inadequately controlled with one dose of methimazole (MMI) and who then become hypothyroid after a minimal dose increase (44). Nine of the clinical studies screened were on the B&R regimen, seven of which were RCT (17, 19–24, 35) and all used titration therapy as the control group.

Three studies including Perozim et al., Jorde et al., and Grebe et al. reported that the B&R regimen had some benefits in the recurrence of Graves’ disease (18, 20, 23). In the study by Perozim et al. (18), 28 patients first received 60 mg of MMI for 4–6 weeks and then received 40 mg of MMI and 75 μg of levothyroxine daily for 18–24 months. After the study was completed, patients were retrospectively divided into a remission group and a non-remission group and were followed up for 2 years. Owing to the low number of cases and the low level of methodological evidence, the study conclusions are debatable. The studies of Jorde et al. (20) and Grebe et al. (23) do not support the B&R regimen reducing the long-term relapse rate of patients with Graves’ disease, but the results show that it can reduce the relapse rate in the short term. It is worth noting that compared with the 12–24 months medication cycle of other clinical studies, these two studies discontinued the drug after 6 months of medication and started a 3-year follow-up. It is not clear whether this short-term clinical benefit is related to the duration of medication. It is possible that a too short medication time may have a more obvious negative impact on the clinical effect of the titration therapy.

In four other clinical studies, including Edmonds and Tellez, McIver et al., Lucas et al., and Nedrebo et al., the results showed that the B&R regimen for the recurrence rate of Graves’ disease was not superior to titration therapy (19, 21, 22, 24). There were differences in the timing of thyroxine addition in these four studies. Edmonds and Tellez (19) and Lucas et al. (22) compared the B&R regimen with the titration therapy, whereas in the studies by McIver et al. (21) and Nedrebo et al. (24), patients received thyroxine for 12–18 months after completing the B&R regimen. The results show that the length of time of the thyroxine addition to the B&R therapy does not reduce the recurrence rate of Graves’ disease and may have more side effects. The remaining two studies focused on other effects of the B&R regimen in the treatment of Graves’ disease. Bromberg et al. compared the effects of the B&R regimen and titration therapy on Graves’ ophthalmopathy, and the results showed no significant difference in the improvement of Graves’ ophthalmopathy (17). Vaidya et al. retrospectively analyzed the medical records of 450 patients with Graves’ disease who had received the B&R regimen or titration regimen, but there was little evidence that the B&R regimen could stabilize patients’ thyroid function (25).

Therefore, the included studies showed that thyroxine addition during the B&R regimen or after stopping the ATD did not show more benefits than the titration regimen in the long-term recurrence rate of Graves’ disease.

#### Thyroxine Addition in the Titration Regimen

The titration regimen is widely used in clinical practice. Throughout the course of the treatment, the dose of ATD is adjusted according to the condition and thyroid hormone concentrations, which can generally be divided into initial, decrement, and maintenance phases. There were 11 RCTs (26, 28–37) among the included studies focused on adding thyroxine to titration therapy.

Among the 11 studies, only the study by Hashizume et al. showed a positive effect (26). The researchers believed that the addition of exogenous thyroxine can inhibit TSH secretion and reduce TSH receptor antibody (TRAb) levels and ultimately reduce the recurrence rate of Graves’ disease. In this study, 109 patients received MMI 30 mg daily for six months until their thyroid function was normal. Then, the experimental group and the control group were administered thyroxine or placebo while receiving the maintenance dose of MMI. After 1 year of combination therapy, MMI was discontinued, but the patients continued with either thyroxine or placebo. The results showed that 1 patient (1.7%) in the thyroxine group and 17 patients (34.7%) in the placebo group had relapsed hyperthyroidism within 3 years after MMI discontinuation.

These results have not been confirmed in subsequent studies, and all the remaining nine studies drew contrary conclusions. Similar to the study by Hashizume et al. (26), in the study by Hoermann et al. (34), thyroxine was added after completion of the titration therapy (34). In this study, patients who successfully underwent ATD treatment for 12–15 months were stratified for risk factors and randomly assigned to receive TSH inhibitor doses of levothyroxine for 2 years or no treatment. The study by Tamai et al. not only compared the difference in the efficacy of titration therapy with or without the addition of thyroxine but also divided patients into two subgroups based on normal or suppressed serum TSH concentrations (28). During the two-year observation period, there was no significant difference in the recurrence rates between the three treatment options. Liu et al. and Yang et al. added thyroxine during the initial or maintenance phases of the titration therapy and discontinued ATD and thyroxine after the treatment (36, 37). Compared with the 18-month treatment cycle of Tamai et al. (28) and Yang et al. (37), the medication cycle used by Liu et al. (36) was 6 months only, quite different from the standard treatment of Graves’ disease. The studies by Pfeilschifter and Ziegler, Rittmaster et al., Raber et al., Glinoer et al., and Wu et al. had longer treatment times (18–36 months), thyroxine was added during the titration process, and the titration therapy was completed (29–33). Even with such long-term thyroxine additions, no benefit was seen in the relapse or remission rate. It should be pointed out that the studies by Liu et al. (36), Yang et al. (37), Glinoer et al. (32), and Wu et al. (33) did not emphasize what dose of thyroxine was needed to inhibit the serum TSH concentration during treatment, which was quite different from Hashizume et al. (26). Therefore, the duration of the thyroxine treatment and whether it inhibits the serum TSH concentration had no effect on the remission or relapse rates of Graves’ disease.

#### Thyroxine Addition in Special Populations

The treatment of Graves’ disease in special groups, such as children and pregnant women, has its special features. In the study by Hashizume et al., 78 patients with Graves’ disease received MMI for 1–3 years before pregnancy and thyroid function remained normal during the first two months of pregnancy (38). MMI was discontinued 5–6 months after the start of the pregnancy and the patients were divided into two groups. Group A was given thyroxine (100 μg/day), and group B was given no drugs from 5 months after the start of the pregnancy to 1 year after delivery. The results showed that thyroxine administration during pregnancy and after delivery can effectively reduce TRAb levels and prevent postpartum recurrence of hyperthyroidism. It is now generally accepted that ATD combined with thyroxine is not recommended during pregnancy, because the side effects of high-dose ATD are more serious in pregnant women (45). Because there are only few studies on this topic, it is unclear whether adding thyroxine during pregnancy can obtain the same clinical benefits as the above studies.

Parents generally consider non-pharmacological treatments for children with Graves’ disease because of the poor compliance or severe side effects caused by ATD. Due to concerns about post-intervention complications, non-pharmacological treatments are often not accepted by children’s parents when the patient is very young (46). Vigone et al. retrospectively studied the effects of a medium dose of ATD with levothyroxine (L-T4) versus monotherapy with ATD in pediatric patients with unstable Graves’ disease (39). The results showed that ATD with L-T4 did not increase the remission rate. However, this combination therapy may be useful when it is difficult to control the thyroid function using ATD therapy alone, and it may be a treatment option to defer certain treatments to the appropriate age.

Although such studies are few and insufficient as clinical evidence, they can also provide some basis for clinical treatment and further research.

### Thyroxine Addition in the Non-Pharmacological Treatment of Graves’ Disease

Four of the clinical studies screened were on the non-pharmacological treatment of Graves’ disease, two of which were RCT (40, 43). Non-pharmacological therapies for Graves’ disease mainly include radioactive iodine 131 (RAI) and surgical treatment.

Taïeb et al. randomly divided 94 patients with Graves**’** disease into group A (50 µg L-T4 daily starting 15 days post-radioiodine and the dose of L-T4 was adapted to the thyroid function test results at 1, 3, and 6 months) or group B (follow-up every 4 weeks and treated with L-T4 when needed), and the follow-up period was 6 months (43). The results indicated that L-T4 administered early after RAI may have potential benefits to patients**’** quality of life, but the optimal strategy for L-T4 dosage and timing remained to be determined. Tallstedt et al. retrospectively analyzed the clinical data of 492 patients who received RAI treatment, divided in 2 groups (41). One group of patients was administered thyroxine prophylactically 2 weeks after the RAI treatment, while the other group was administered thyroxine only when hypothyroidism occurred. After 18 months of follow-up, it was shown that early thyroxine administration after RAI treatment can reduce the occurrence of Graves**’** ophthalmopathy. The remaining two studies were related to the role of thyroxine in thyroid surgery.

Kaur et al. randomly assigned 34 patients with Graves**’** disease to three treatment groups, first using ATD to normalize thyroid function, and then supplementing with thyroxine (40). The first group of patients stopped ATD and thyroxine treatment 10 days before partial thyroidectomy and received only Lugol**’**s iodine treatment. In the second group, only ATD and thyroxine were used for treatment until surgery. In the third group, ATD and thyroxine continued until the day of surgery, and patients also received Lugol**’**s iodine treatment for 10 days. The main role of iodine is to inhibit the release of thyroxine from the thyroid gland, which is often used in preparations for thyroidectomy. The results of the study showed that in patients who had used ATD and thyroxine to normalize the thyroid function, preoperative iodine therapy had no effect on the blood vessels of the gland, blood loss from surgery, or size of the thyroid follicles. Zhu et al. divided 471 patients with hyperthyroidism into an experimental group and a control group (42). The control group was given ATD and iodine preparations before surgery, while the experimental group was further divided into four subgroups and treated with **“**sequential thyroid defunctionalization followed by thyroxine supplementation.**”** The four subgroups received different doses of ATD and thyroxine at different time periods. The results showed that **“**sequential thyroid defunctionalization followed by thyroxine supplementation**”** could effectively reduce the bleeding volume and the incidence of postoperative complications of partial thyroidectomy.

From these results, some clinical benefits could be seen, but these studies were small in size and scattered in purpose. If these conclusions are to be confirmed, high-quality clinical research is needed.

## Discussion

The relative advantages and disadvantages of various treatments for Graves’ disease are still controversial. Each method is effective, but none is perfect. The choice depends on the treating physician, and increasingly on the wishes of the patient and local medical resources. Therefore, it is necessary to study the clinical efficacy of various treatments, including remission/relapse rate, quality of life and so on, which can provide the basis for the choice of treatment.

ATD have been part of the standard treatment for Graves’ disease for many years and may lead to long-term remission. Searching through the results, we found two modes of combined use of oral ATD and thyroxine (1): the B&R regimen, that is, simultaneous use with high doses of ATD (2); adding thyroid in the titration regimen. It has long been adopted by many researchers that the recurrence of hyperthyroidism caused by Graves’ disease or the interruption of the ATD treatment is associated with elevated TRAb levels in most patients. Earlier research by Hashizume et al. (26). suggested that exogenous thyroxine supplementation after ATD reduction and discontinuation could inhibit TSH secretion and reduce the thyroid-stimulating antibody (TSAb) production, reducing the recurrence of Graves’ disease. The same findings were achieved in subsequent studies in pregnant women (26). However, the results of most subsequent studies in various countries showed that thyroxine addition to maintain normal TSH serum levels or inhibit TSH serum levels did not reduce TSAb levels, and the recurrence rate did not decrease.

A prospective multicenter observational cohort study of 344 patients showed that the prediction of biological euthyroism during treatment with antithyroid drugs is higher during titration compared to B&R regime. Whether one regimen is clinically more advantageous than the other remains unclear (47). With regard to the huge differences in the results of these studies, existing explanations point to possible genetic and iodine intake differences (48, 49). TRAb is due to an uncertain mechanism that changes the body’s immune homeostasis (50–52). There is evidence that ATD has an immunosuppressive effect and may help suppress the production of TRAb, but this is still controversial (53, 54). A significant reduction in TRAb can be observed in patients receiving ATD; however, TRAb may continue to increase in many patients after the long-term treatment achieves remission of hyperthyroidism (51). Evidence of a dose-dependent immunosuppressive effect of MMI on the intensity of autoimmune processes in the thyroid gland has not been found (55). It is now widely accepted that the relief of Graves’ disease relates to the restoration of normal thyroid function and is not a special drug effect. This conclusion underlines the importance of maintaining a normal thyroid in patients with Graves’ disease (56). Since thyroxine combined with ATD is more complicated than monotherapy, and from most research results, this combination therapy does not make thyroid function more stable. Moreover, the B&R regimen has produced conflicting results in terms of the possibility of disease remission and side effects (57). Most of the included studies did not adequately report side effects. In studies that mentioned side effects (19, 20, 23, 25), the side effects of B&R regimen were more than those of titration regimen. From our research results, thyroxine combined with ATD cannot obtain a more stable thyroid function, higher remission rates and lower recurrence rates than ATD alone. However, in some patients treated with ATD who have poor thyroid function control, the addition of thyroxine can be tried. In the studies we reviewed, we also observed some positive effects, such as the benefits on bone metabolism in elderly patients (37), the benefits on goiter (29), etc. However, these observations need to be confirmed in further clinical trials.

These clinical trials have shown some benefits in adding thyroxine to the surgical treatment of Graves’ disease. Recurrent laryngeal nerve injury, parathyroid injury, and postoperative bleeding are serious complications of thyroid surgery and are closely related to the adequacy of preoperative preparation. Preoperative preparation for thyroid surgery, including ATD use to restore thyroid function, usually requires the use of a saturated potassium iodide solution to reduce the blood flow to the thyroid gland to limit the perioperative blood loss (58). However, there are some disadvantages. First, taking iodine for a long time or abruptly stopping it will cause a sudden increase in free thyroid hormone and cause thyroid crisis. Second, although the size of the thyroid gland and the blood supply can be reduced, the glandular tissue become hard and brittle, and there is significant surgical bleeding. In addition, iodine preparations are not appropriate for patients allergic to iodine. Using the “preoperative hypothyroidism and then add thyroxine” preoperative preparation method, exogenous thyroxine can effectively reduce thyroid congestion and swelling, decrease thyroid blood supply, soften thyroid tissue, and make the thyroid gland similar to the normal thyroid gland, thus allowing for a more convenient surgery and fewer surgical complications (42, 59).

A study of 2,430 patients with Graves’ disease followed for 6 to 10 years showed that only 35.7% of all patients reached a normal thyroid hormone status without levothyroxine replacement (60). Hypothyroidism is common after Graves’ disease undergoes RAI and surgery. The results of the included studies showed that an early use of thyroxine after RAI could not significantly reduce the incidence of severe hypothyroidism but could reduce the incidence of Graves’ ophthalmopathy and improve the patients’ quality of life. Studies have shown that after RAI treatment of Graves’ disease, the incidence of ophthalmopathy is worse than after oral ATD (61). Elevated TSH levels are positively associated with the development of Graves’ ophthalmopathy, and thyroxine addition may enhance TSH inhibition after RAI (62, 63).

In addition, there are many meaningful case reports showing the use of thyroxine in Graves’ disease. ATD overuse in pregnant women with Graves’ disease can cause hypothyroidism in fetuses, and intraamniotic injections of levothyroxine have been proven successful in fetal treatment (64).

In summary, the available evidence does not support indiscriminate thyroxine supplementation in the treatment of Graves’ disease, especially when used in combination with oral antithyroid drugs. The focus of future research is to further clinical studies to explore the indications for adding thyroxine to the treatment of Graves’ disease.

## Data Availability Statement

The original contributions presented in the study are included in the article/**Supplementary Material**, further inquiries can be directed to the corresponding authors.

## Author Contributions

JL and LB designed the work of review. JL, LB, and FW reviewed the literature available on this topic and wrote the paper. MW, YX, and WY contributed to the scientific writing of the manuscript. JL, LB, and JW revised the manuscript. All authors approved the paper for publication. JL, LB, FW, MW, YX, and WY contributed equally to this work. All authors contributed to the article and approved the submitted version.

## Conflict of Interest

The authors declare that the research was conducted in the absence of any commercial or financial relationships that could be construed as a potential conflict of interest.

## References

[1] Hollowell JG Staehling NW Flanders WD Hannon WH Gunter EW Spencer CA Serum TSH, T(4), and thyroid antibodies in the United States population (1988 to 1994): National Health and Nutrition Examination Survey (NHANES III). J Clin Endocrinol Metab (2002) 87(2):489–99. 10.1210/jcem.87.2.8182 11836274

[2] Garmendia Madariaga A Santos Palacios S Guillen-Grima F Galofre JC The incidence and prevalence of thyroid dysfunction in Europe: a meta-analysis. J Clin Endocrinol Metab (2014) 99(3):923–31. 10.1210/jc.2013-2409 24423323

[3] Taylor PN Albrecht D Scholz A Gutierrez-Buey G Lazarus JH Dayan CM Global epidemiology of hyperthyroidism and hypothyroidism. Nat Rev Endocrinol (2018) 14(5):301–16. 10.1038/nrendo.2018.18 29569622

[4] Zimmermann MB Jooste PL Pandav CS Iodine-deficiency disorders. Lancet (2008) 372(9645):1251–62. 10.1016/S0140-6736(08)61005-3 18676011

[5] Taylor PN Okosieme OE Dayan CM Lazarus JH Therapy of endocrine disease: Impact of iodine supplementation in mild-to-moderate iodine deficiency: systematic review and meta-analysis. Eur J Endocrinol (2014) 170(1):R1–r15. 10.1530/EJE-13-0651 24088547

[6] Laurberg P Pedersen KM Vestergaard H Sigurdsson G High incidence of multinodular toxic goitre in the elderly population in a low iodine intake area vs. high incidence of Graves’ disease in the young in a high iodine intake area: comparative surveys of thyrotoxicosis epidemiology in East-Jutland Denmark and Iceland. J Intern Med (1991) 229(5):415–20. 10.1111/j.1365-2796.1991.tb00368.x 2040867

[7] Marino M Latrofa F Menconi F Chiovato L Vitti P Role of genetic and non-genetic factors in the etiology of Graves’ disease. J Endocrinol Invest (2015) 38(3):283–94. 10.1007/s40618-014-0214-2 25421156

[8] Chiovato L Pinchera A Stressful life events and Graves’ disease. Eur J Endocrinol (1996) 134(6):680–2. 10.1530/eje.0.1340680 8766933

[9] Wiersinga WM Smoking and thyroid. Clin Endocrinol (Oxf) (2013) 79(2):145–51. 10.1111/cen.12222 23581474

[10] Menconi F Marcocci C Marino M Diagnosis and classification of Graves’ disease. Autoimmun Rev (2014) 13(4-5):398–402. 10.1016/j.autrev.2014.01.013 24424182

[11] Vita R Lapa D Vita G Trimarchi F Benvenga S A patient with stress-related onset and exacerbations of Graves disease. Nat Clin Pract Endocrinol Metab (2009) 5(1):55–61. 10.1038/ncpendmet1006 19029994

[12] Vita R Lapa D Trimarchi F Vita G Fallahi P Antonelli A Certain HLA alleles are associated with stress-triggered Graves’ disease and influence its course. Endocrine (2017) 55(1):93–100. 10.1007/s12020-016-0909-6 26951052

[13] Topcu CB Celik O Tasan E Effect of stressful life events on the initiation of Graves’ disease. Int J Psychiatry Clin Pract (2012) 16(4):307–11. 10.3109/13651501.2011.631016 22136213

[14] Burch HB Burman KD Cooper DS A 2011 survey of clinical practice patterns in the management of Graves’ disease. J Clin Endocrinol Metab (2012) 97(12):4549–58. 10.1210/jc.2012-2802 23043191

[15] Francis N Francis T Lazarus JH Okosieme OE Current controversies in the management of Graves’ hyperthyroidism. Expert Rev Endocrinol Metab (2020) 15(3):159–69. 10.1080/17446651.2020.1754192 32315207

[16] Abraham P Avenell A McGeoch SC Clark LF Bevan JS Antithyroid drug regimen for treating Graves’ hyperthyroidism. Cochrane Database Syst Rev (2010) 2010(1):CD003420. 10.1002/14651858.CD003420.pub4 PMC6599817 20091544

[17] Bromberg N Romaldini JH Werner RS Sgarbi JA Werner MC The evolution of Graves’ ophthalmopathy during treatment with antithyroid drug alone and combined with triiodothyronine. J Endocrinol Invest (1992) 15(3):191–5. 10.1007/BF03348703 1624679

[18] Perozim LM Lima N Knobel M Cavaliere H Medeiros-Neto G Treatment of Graves’ disease: effects of the administration of L-thyroxine associated with methimazole as a single daily dose. Eur J Med (1993) 2(2):70–4. 8258020

[19] Edmonds CJ Tellez M Treatment of Graves’ disease by carbimazole: high dose with thyroxine compared to titration dose. Eur J Endocrinol (1994) 131(2):120–4. 10.1530/eje.0.1310120 8075780

[20] Jorde R Ytre-Arne K Stormer J Sundsfjord J Short-term treatment of Graves’ disease with methimazole in high versus low doses. J Internal Medicine (1995) 238(2):161–5. 10.1111/j.1365-2796.1995.tb00914.x 7629484

[21] McIver B Rae P Beckett G Wilkinson E Gold A Toft A Lack of effect of thyroxine in patients with Graves’ hyperthyroidism who are treated with an antithyroid drug. N Engl J Med (1996) 334(4):220–4. 10.1056/NEJM199601253340403 8531998

[22] Lucas A Salinas I Rius F Pizarro E Granada ML Foz M Medical therapy of Graves’ disease: does thyroxine prevent recurrence of hyperthyroidism? J Clin Endocrinol Metab (1997) 82(8):2410–3. 10.1210/jcem.82.8.4118 9253309

[23] Grebe SKG Feek CM Ford HC Fagerström JN Cordwell DP Delahunt JW A randomized trial of short-term treatment of Graves’ disease with high- dose carbimazole plus thyroxine versus low-dose carbimazole. Clin Endocrinol (1998) 48(5):585–92. 10.1046/j.1365-2265.1998.00446.x 9666870

[24] Nedrebo BG Holm P II Uhlving S Sorheim J II Skeie S Eide GE Predictors of outcome and comparison of different drug regimens for the prevention of relapse in patients with Graves’ disease. Eur J Endocrinol (2002) 147(5):583–9. 10.1530/eje.0.1470583 12444889

[25] Vaidya B Wright A Shuttleworth J Donohoe M Warren R Brooke A Block & replace regime versus titration regime of antithyroid drugs for the treatment of Graves’ disease: a retrospective observational study. Clin Endocrinol (Oxf) (2014) 81(4):610–3. 10.1111/cen.12478 24801484

[26] Hashizume K Ichikawa K Sakurai A Suzuki S Takeda T Kobayashi M Administration of thyroxine in treated Graves’ disease. Effects on the level of antibodies to thyroid-stimulating hormone receptors and on the risk of recurrence of hyperthyroidism. N Engl J Med (1991) 324(14):947–53. 10.1056/NEJM199104043241403 1900575

[27] Kuo SW Huang WS Hu CA Liao WK Fung TC Wu SY Effect of thyroxine administration on serum thyrotropin receptor antibody and thyroglobulin levels in patients with Graves’ hyperthyroidism during antithyroid drug therapy. Eur J Endocrinol (1994) 131(2):125–30. 10.1530/eje.0.1310125 8075781

[28] Tamai H Hayaki I Kawai K Komaki G Matsubayashi S Kuma K Lack of effect of thyroxine administration on elevated thyroid stimulating hormone receptor antibody levels in treated Graves’ disease patients. J Clin Endocrinol Metab (1995) 80(5):1481–4. 10.1210/jcem.80.5.7744989 7744989

[29] Pfeilschifter J Ziegler R Suppression of serum thyrotropin with thyroxine in patients with Graves’ disease: effects on recurrence of hyperthyroidism and thyroid volume. Eur J Endocrinol (1997) 136(1):81–6. 10.1530/eje.0.1360081 9037131

[30] Rittmaster RS Abbott EC Douglas R Givner ML Lehmann L Reddy S Effect of methimazole, with or without L-thyroxine, on remission rates in Graves’ disease. J Clin Endocrinol Metab (1998) 83(3):814–8. 10.1210/jcem.83.3.4613 9506733

[31] Raber W Kmen E Waldhäusl W Vierhapper H Medical therapy of Graves’ disease: effect on remission rates of methimazole alone and in combination with triiodothyronine. Eur J Endocrinol (2000) 142(2):117–24. 10.1530/eje.0.1420117 10664518

[32] Glinoer D de Nayer P Bex M Belgian Collaborative Study Group on Graves’ Disease Effects of l-thyroxine administration, TSH-receptor antibodies and smoking on the risk of recurrence in Graves’ hyperthyroidism treated with antithyroid drugs: a double-blind prospective randomized study. Eur J Endocrinol (2001) 144(5):475–83. 10.1530/eje.0.1440475 11331213

[33] Wu G Jie Y Situ Y [Effect of thyroxine upon prevention of recurrence of Graves’ disease treated with antithyroid drugs]. Zhonghua Yi Xue Za Zhi (2001) 81(5):274–5. CNKISUNZHYX.0.2001-05-006 11798886

[34] Hoermann R Quadbeck B Roggenbuck U Szabolcs I Pfeilschifter J Meng W Relapse of Graves’ disease after successful outcome of antithyroid drug therapy: results of a prospective randomized study on the use of levothyroxine. Thyroid (2002) 12(12):1119–28. 10.1089/105072502321085225 12593726

[35] Mastorakos G Doufas AG Mantzos E Mantzos J Koutras DA T4 but not T3 administration is associated with increased recurrence of Graves’ disease after successful medical therapy. J Endocrinol Invest (2003) 26(10):979–84. 10.1007/BF03348195 14759070

[36] Liu X Qiang W Liu X Liu L Liu S Gao A A 6-year follow-up of a randomized prospective trial comparing methimazole treatment with or without exogenous L-thyroxine in Chinese patients with Graves’ disease. Exp Clin Endocrinol Diabetes (2014) 122(10):564–7. 10.1055/s-0034-1377045 25140995

[37] Yang F Yu TT Jiang XL Li J Wang J Lei XY Coupling methimazole with L-thyroxine on bone of older patients with Graves’ disease. Int J Clin Exp Medicine (2016) 9(9):18458–64.

[38] Hashizume K Ichikawa K Nishii Y Kobayashi M Sakurai A Miyamoto T Effect of administration of thyroxine on the risk of postpartum recurrence of hyperthyroid Graves’ disease. J Clin Endocrinol Metab (1992) 75(1):6–10. 10.1210/jcem.75.1.1642700 1642700

[39] Vigone MC Peroni E Di Frenna M Mora S Barera G Weber G “Block-and-replace” treatment in Graves’ disease: experience in a cohort of pediatric patients. J Endocrinol Invest (2019) 43:595–600. 10.1007/s40618-019-01144-0 31713721

[40] Kaur S Parr JH Ramsay ID Hennebry TM Jarvis KJ Lester E Effect of preoperative iodine in patients with Graves’ disease controlled with antithyroid drugs and thyroxine. Ann R Coll Surg Engl (1988) 70(3):123–7. PMC2498739 2457351

[41] Tallstedt L Lundell G Blomgren H Bring J Does early administration of thyroxine reduce the development of Graves’ ophthalmopathy after radioiodine treatment? Eur J Endocrinol (1994) 130(5):494–7. 10.1530/eje.0.1300494 8180678

[42] Zhu JQ Li ZH Gong RX Wei T Zhang H Zhang WY Sequential defunctionalization followed by thyroxine supplementation as preoperative preparation of hyperthyroid patients undergoing thyroidectomy. Chin Med J (2008) 121(20):2010–5. 10.1097/00029330-200810020-00012 19080266

[43] Taïeb D Bournaud C Eberle MC Catargi B Schvartz C Cavarec MB Quality of life, clinical outcomes and safety of early prophylactic levothyroxine administration in patients with Graves’ hyperthyroidism undergoing radioiodine therapy: a randomized controlled study. Eur J Endocrinol (2016) 174(4):491–502. 10.1530/EJE-15-1099 26772985

[44] Ross DS Burch HB Cooper DS Greenlee MC Laurberg P Maia AL 2016 American Thyroid Association Guidelines for Diagnosis and Management of Hyperthyroidism and Other Causes of Thyrotoxicosis. Thyroid (2016) 26(10):1343–421. 10.1089/thy.2016.0229 27521067

[45] Alexander EK Pearce EN Brent GA Brown RS Chen H Dosiou C 2017 Guidelines of the American Thyroid Association for the Diagnosis and Management of Thyroid Disease During Pregnancy and the Postpartum. Thyroid (2017) 27(3):315–89. 10.1089/thy.2016.0457 28056690

[46] Rivkees SA Controversies in the management of Graves’ disease in children. J Endocrinol Invest (2016) 39(11):1247–57. 10.1007/s40618-016-0477-x 27153850

[47] Žarković M Wiersinga W Perros P Bartalena L Donati S Okosieme O Antithyroid drugs in Graves’ hyperthyroidism: differences between “block and replace” and “titration” regimes in frequency of euthyroidism and Graves’ orbitopathy during treatment. J Endocrinol Invest (2020). 10.1007/s40618-020-01320-7 32524368

[48] Ishizuki Y Hirooka Y Murata Y Urinary Iodide Excretion in Japanese People and Thyroid Dysfunction. Folia Endocrinol Japonica (1992) 68(5):550–6. 10.1507/endocrine1927.68.5_550 1644207

[49] Lee SM Lewis J Buss DH Holcombe GD Lawrance PR Iodine in British foods and diets. Br J Nutr (1994) 72(3):435–46. 10.1079/bjn19940045 7947658

[50] Onaya T Kotani M Yamada T Ochi Y New in vitro tests to detect the thyroid stimulator in sera from hyperthyroid patients by measuring colloid droplet formation and cyclic AMP in human thyroid slices. J Clin Endocrinol Metab (1973) 36(5):859–66. 10.1210/jcem-36-5-859 4349046

[51] Fenzi G Hashizume K Roudebush CP DeGroot LJ Changes in thyroid-stimulating immunoglobulins during antithyroid therapy. J Clin Endocrinol Metab (1979) 48(4):572–6. 10.1210/jcem-48-4-572 581875

[52] Rees Smith B McLachlan SM Furmaniak J Autoantibodies to the thyrotropin receptor. Endocr Rev (1988) 9(1):106–21. 10.1210/edrv-9-1-106 3286231

[53] Weetman AP McGregor AM Hall R Evidence for an effect of antithyroid drugs on the natural history of Graves’ disease. Clin Endocrinol (Oxf) (1984) 21(2):163–72. 10.1111/j.1365-2265.1984.tb03456.x 6205795

[54] Wenzel KW Lente JR Similar effects of thionamide drugs and perchlorate on thyroid-stimulating immunoglobulins in Graves’ disease: evidence against an immunosuppressive action of thionamide drugs. J Clin Endocrinol Metab (1984) 58(1):62–9. 10.1210/jcem-58-1-62 6196375

[55] Escobarmorreale HF Methimazole has no dose-related effect on the serum concentrations of soluble class I major histocompatibility complex antigens, soluble interleukin-2 receptor, and beta 2-microglobulin in patients with Graves’ disease. Thyroid (1996) 6(6):29–36. 10.1089/thy.1996.6.29 8777381

[56] Laurberg P Remission of Graves’ disease during anti-thyroid drug therapy. Time to reconsider the mechanism? Eur J Endocrinol (2006) 155(6):783–6. 10.1530/eje.1.02295 17132745

[57] Cheetham TD Hughes I Barnes ND Wraight EP Treatment of hyperthyroidism in young people. Arch Dis Childhood (1998) 78(3):207–9. 10.1136/adc.78.3.207 PMC1717503 9613348

[58] Erbil Y Ozluk Y Giriş M Salmaslioglu A Issever H Barbaros U Effect of Lugol Solution on Thyroid Gland Blood Flow and Microvessel Density in the Patients with Graves’ Disease. J Clin Endocrinol Metab (2007) 92(6):2182–9. 10.1210/jc.2007-0229 17389702

[59] Bartalena L Diagnosis and management of Graves disease: a global overview. Nat Rev Endocrinol (2013) 9(12):724–34. 10.1038/nrendo.2013.193

[60] Sjölin G Holmberg M Törring O Byström K Khamisi S de Laval D The Long-Term Outcome of Treatment for Graves’ Hyperthyroidism. Thyroid (2019) 29(11):1545–57. 10.1089/thy.2019.0085 31482765

[61] Bartalena L Marcocci C Bogazzi F Manetti L Tanda ML Dell'Unto E Relation between therapy for hyperthyroidism and the course of Graves’ ophthalmopathy. N Engl J Med (1998) 338(2):73–8. 10.1056/NEJM199801083380201 9420337

[62] Tengroth B Endocrine exophthalmos: effects of thyrotropin preparations and the thyroxin isomers: quantitative evaluations in guinea pigs. Acta Ophthalmol Suppl. (1961) Suppl 65: (65):1. 13775892

[63] Almqvist S Algvere P HYPOTHYROIDISM IN PROGRESSIVE OPHTHALMOPATHY OF GRAVES’ DISEASE. Acta Ophthalmol (Copenh) (1972) 50(6):761–70. 10.1111/j.1755-3768.1972.tb06615.x 4678867

[64] Miyata I Abe-Gotyo N Tajima A Yoshikawa H Teramoto S Seo M Successful intrauterine therapy for fetal goitrous hypothyroidism during late gestation. Endocr J (2007) 54(5):813–7. 10.1507/endocrj.k07-047 17917306

