# Supplementary figures and images for: Effect of Addition of Thyroxine in the Treatment of Graves’ Disease: A Systematic Review
Source: Front Endocrinol (Lausanne). 2021 Jan 25;11:560157. doi: 10.3389/fendo.2020.560157 (PMC7868565; doi:10.3389/fendo.2020.560157)

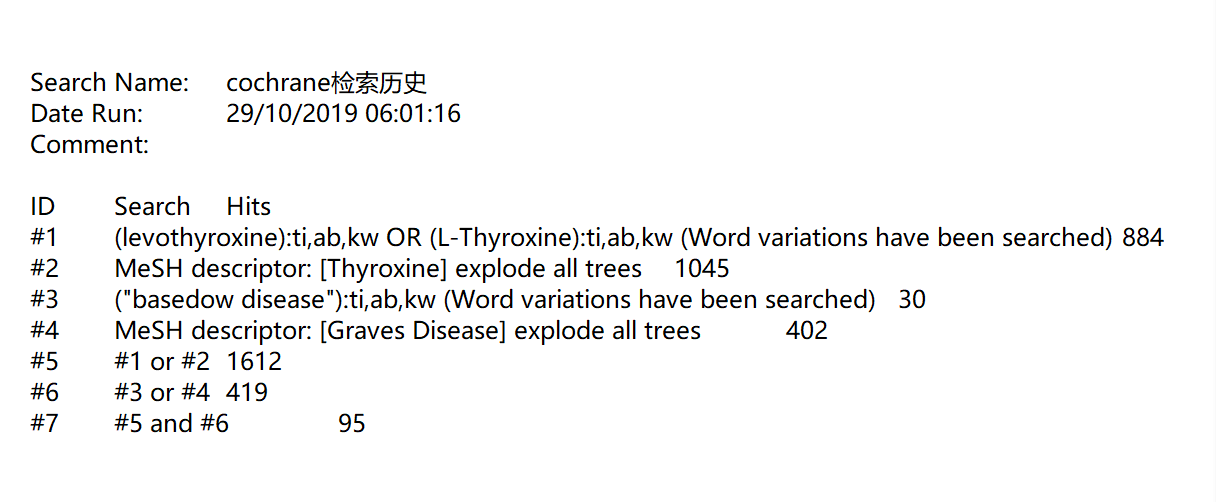

Supplement: Supplementary file 1 [file Image_1.png]

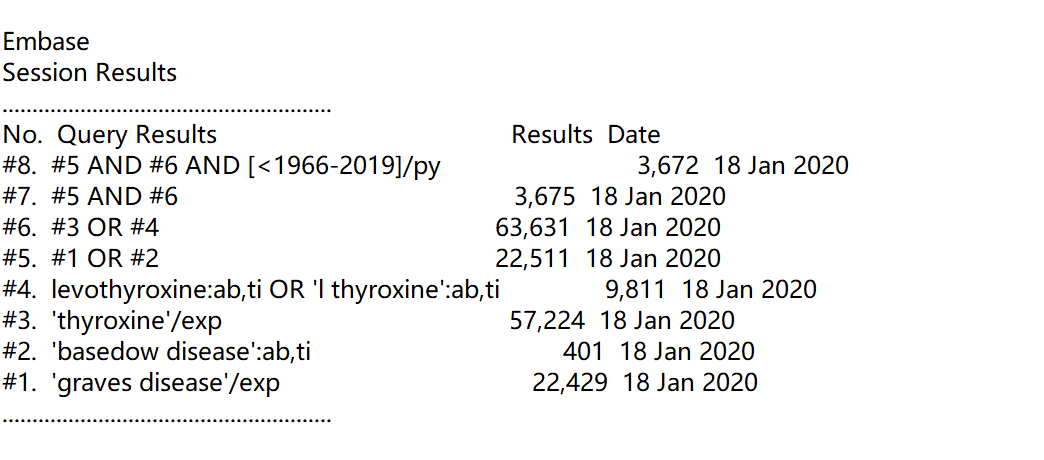

Supplement: Supplementary file 2 [file Image_2.png]

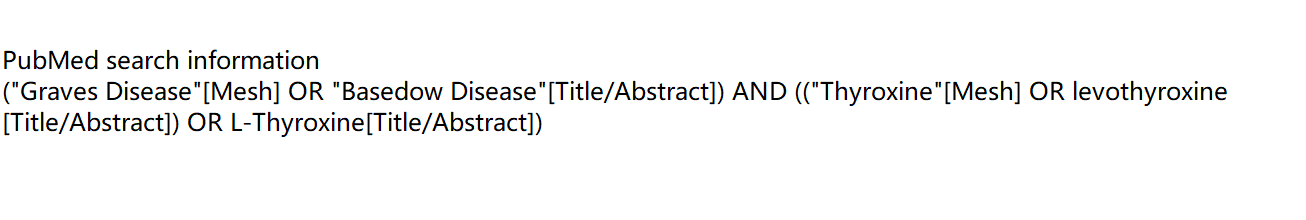

Supplement: Supplementary file 3 [file Image_3.png]
